# The Effects of Bariatric Surgery on Female Fertility: A Narrative Review

**DOI:** 10.3390/ijms27083665

**Published:** 2026-04-20

**Authors:** Maria Iliopoulou, Theoharis Papageorgiou, Makarios Eleftheriadis, George Mastorakos, Georgios Valsamakis

**Affiliations:** 1 Unit of Endocrinology, Aretaieio University Hospital, Athens Medical School, National and Kapodistrian University of Athens, 11528 Athens, Greece; mariahelip@yahoo.gr (M.I.); mastorakg@gmail.com (G.M.); 2 Iaso Maternity Hospital, IVF Unit Institute of Life—IASO, 15123 Athens, Greece; theoharispapageorgiou@gmail.com; 3 Department of Obstetrics and Gynecology, Aretaieio University Hospital, Athens Medical School, National and Kapodistrian University of Athens, 11528 Athens, Greece; melefth@med.uoa.gr

**Keywords:** bariatric surgery, obesity, female fertility, insulin resistance, polycystic ovary syndrome, pregnancy, sleeve gastrectomy, Roux-en-Y gastric bypass

## Abstract

Obesity is associated with menstrual dysfunction, anovulation, and infertility, particularly in women with polycystic ovary syndrome (PCOS). This narrative review summarizes evidence on the effects of bariatric surgery [focusing on sleeve gastrectomy (SG) Roux-en-Y gastric bypass (RYGB)] on female reproductive function and fertility outcomes. Developed according to SANRA (Scale for the Assessment of Narrative Review Articles) principles, a structured search of PubMed, Scopus, and Web of Science (English language; inception–30 September 2025) was conducted, using fertility-related terms (e.g., fertility, ovulation, IVF/ART, AMH, PCOS, pregnancy, live birth, time to conception) combined with bariatric surgery terms (SG/VSG, RYGB, metabolic/bariatric surgery, and weight loss surgery). Guidelines from IFSO, BOMSS, and ASMBS were also reviewed. Findings were synthesized narratively. Across mainly observational studies, bariatric surgery is associated with improved menstrual regularity, increased ovulation, reduced hyperandrogenism, and improved insulin sensitivity, with higher conception rates reported after substantial weight loss. AMH responses are inconsistent across studies and their clinical significance remains uncertain. SG and RYGB appear to improve fertility-related outcomes in women with obesity. Programming of pregnancy and nutritional monitoring are critical. In conclusion, long-term, standardized reproductive endpoints are needed to clarify bariatric surgery-associated effects during pregnancy.

## 1. Introduction

The global prevalence of obesity increased markedly, with estimates indicating an almost threefold rise between 1975 and 2016. In 2016, approximately 1.9 billion adults (≥18 years) met criteria for obesity (BMI 25.0–29.9 kg/m^2^), corresponding to 40% of women and 39% of men, while about 650 million individuals were classified as having obesity (BMI ≥ 30 kg/m^2^), including 11% of men and 15% of women [1,2]. Obesity is defined as a chronic disease characterized by excessive adipose tissue accumulation, which contributes to pathophysiological processes, and is operationally defined as a BMI > 30 kg/m^2^ [3]. Overweight and obesity constitute major global public health concerns and are associated with multiple comorbidities, including type 2 diabetes mellitus, dyslipidemia, hypertension, coronary heart disease, gallstone disease, and postmenopausal breast and endometrial cancer, as well as reproductive dysfunction in women of reproductive age [4,5]. According to international guidance, bariatric surgery is indicated for individuals with class III obesity (BMI ≥ 40 kg/m^2^) or class II obesity (BMI 35–39 kg/m^2^) in the presence of obesity-related comorbidities [6]. For Asian populations, lower BMI thresholds are recommended; a BMI ≥ 25 kg/m^2^ is considered to represent clinical obesity, and metabolic–bariatric surgery (MBS) should be considered from BMI ≥ 27.5 kg/m^2^ [6].

The American Society for Reproductive Medicine (ASRM) defines infertility as a disease characterized by the inability to conceive after ≥12 months of regular, unprotected intercourse [7]. Estimates from the World Health Organization (WHO) indicate that infertility affects approximately 50–80 million women globally, although diagnostic timelines and evaluation strategies differ for women aged >35 years; these considerations are beyond the scope of the present review [7]. At the couple level, infertility is estimated to affect roughly 70 million couples worldwide [7]. Reported lifetime infertility rates vary by region, ranging from 3.3% to 21.3% in Eastern Mediterranean countries, to approximately 7% in England, and up to 30% in several African settings [8]. Demographic projections incorporating contemporary fertility patterns and treatments suggest that overall population growth may approach zero over the period 2036–2041 [8]. Infertility is increasingly recognized as a major public health and social issue, and its association with obesity appears particularly relevant among women of reproductive age through multiple pathophysiological pathways [9].

Global BMI trends show persistent increases among girls and women across multiple regions, and a substantial proportion of women of reproductive age are affected by obesity [1]. Compared with women of normal weight, women with obesity exhibit reduced fecundity in both spontaneous conception cycles and infertility treatment cycles [10]. This population has also been reported to have higher risks of miscarriage and congenital anomalies [11]. Preconception BMI above 25 kg/m^2^ has been associated with a greater likelihood of delayed conception and adverse pregnancy outcomes, including pregnancy loss and very preterm birth, compared with women with BMI in the 20.0–24.9 kg/m^2^ range [9,12,13]. Conversely, structured weight loss interventions have been associated with improvements in menstrual cyclicity and ovulatory frequency, thereby increasing the probability of conception and successful pregnancy [9].

Management of obesity includes lifestyle-based weight reduction strategies (dietary modification and increased physical activity), anti-obesity pharmacotherapy, and MBS [9,14]. MBS is increasingly utilized for obesity treatment and typically results in >20% total body weight loss [15,16], a magnitude associated with clinically meaningful improvements in PCOS, type 2 diabetes mellitus, and hypertension [17]. Evidence also indicates that MBS is associated with improved fertility and more favorable pregnancy-related outcomes for both mother and offspring [18]. Globally, the most frequently performed bariatric procedures are RYGB and VSG, which together account for approximately 90% of operations [6]. In accordance with the National Institute for Health and Care Excellence (NICE) guidance, bariatric surgery is generally indicated for individuals with BMI > 40 kg/m^2^ or BMI > 35 kg/m^2^ in the presence of significant obesity-related comorbidities (e.g., type 2 diabetes mellitus or hypertension) [6]. In patients with severe obesity, MBS provides the most durable weight loss and is associated with greater cardiometabolic risk reduction than lifestyle intervention or pharmacotherapy alone [19].

Our synthesis aligns with the prior literature showing that bariatric surgery in women with obesity is associated with improved menstrual cyclicity, reduced hyperandrogenism, and a higher likelihood of conception, particularly among those with PCOS [20]. Clinical reviews also emphasize the need to delay conception after surgery and to implement structured nutritional assessment and appropriate nutritional supplementation during pregnancy [21]. Recent reviews have summarized evidence linking obesity and bariatric surgery with female reproductive outcomes, including fertility and postoperative pregnancy management [20,21]. However, clinically important uncertainty remains regarding the procedure-specific effects of SG versus RYGB on reproductive endocrinology, ovarian reserve biomarkers, particularly AMH, and the extent to which postoperative biochemical changes correspond to clinically relevant fertility endpoints. Accordingly, this narrative review aims to summarize current evidence on SG and RYGB in women of reproductive age by integrating mechanistic pathways with clinical outcomes, including menstrual cyclicity, ovulation, time to conception, and pregnancy rates, and by outlining implications for individualized preconception counseling. Specifically, this review examines the effects of bariatric surgery (RYGB and SG/VSG) on female fertility, AMH levels, reproductive hormonal profiles, insulin resistance, ovulatory function, and reproductive outcomes were reviewed.

## 2. Results

### 2.1. Obesity and Infertility in Women

Obesity is a chronic, multifactorial disease defined by excess adipose tissue accumulation, which is associated with increased long-term morbidity and reduced life expectancy [22]. In women, obesity adversely affects fecundity in both spontaneous conception and assisted reproductive technology (ART) settings, with higher rates of infertility and miscarriage, and it is also associated with psychological comorbidity, supporting the need for multidisciplinary and equity-oriented care [7,23]. Reproductive disorders are frequently observed among overweight and obese women of reproductive age [4]. In the United States, obesity affects approximately 36.5% of women aged 20–39 years [22].

Women with obesity demonstrate reduced per-cycle and cumulative probabilities of conception, prolonged time to pregnancy, and lower IVF success rates accompanied by increased miscarriage risk; reported estimates include an approximately 5% decrease in pregnancy probability per BMI unit above 29 kg/m^2^ and a roughly 10% reduction in IVF success among overweight women [16]. These reproductive outcomes are consistent with broader data indicating that higher body weight and weight gain, even within the normal BMI range, are associated with increased coronary heart disease (CHD) risk in women [5].

A systematic review and meta-analysis reported increased odds of psychological distress (OR 1.63) and depression (OR 1.40) among infertile women, and smoking has been associated with nearly a twofold increase in infertility odds [23]. In low-resource settings, infertility may be associated with substantial social harms (including stigma, isolation, and violence), underscoring important equity considerations in reproductive health [7,23].

**Figure 1:**
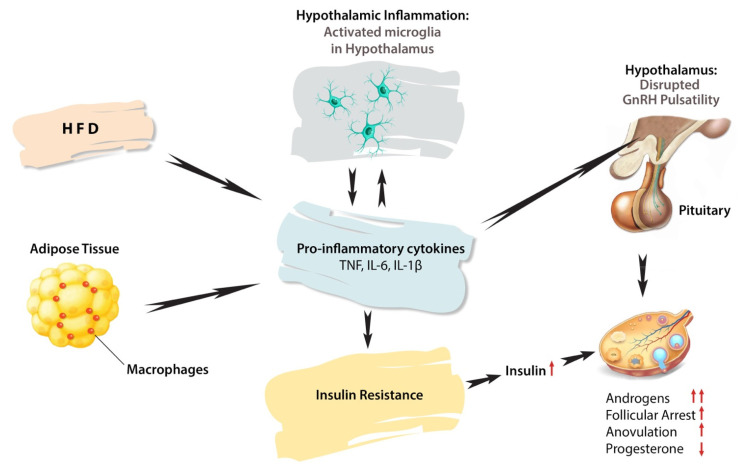
Schematic representation of obesity-associated inflammatory and endocrine mechanisms involved in female reproductive dysfunction. The figure summarizes the proposed interactions between high-fat diet (HFD), adipose tissue-macrophage activation, pro-inflammatory cytokines, hypothalamic inflammation, insulin resistance, and ovarian dysfunction which leads to impaired reproductive outcomes.

Obesity, particularly severe obesity, is associated with clinically relevant disruption of the HPO axis and related manifestations such as hirsutism, menstrual irregularity, endometrial dysfunction, and adverse gestational outcomes, including increased miscarriage risk [16,24,25]. ART outcomes are also adversely affected; women with obesity may produce fewer and less mature oocytes, contributing to lower fertilization rates [26]. Emerging evidence suggests that hypothalamic inflammation may represent an additional mechanistic link between obesity and reproductive dysfunction, particularly in women with PCOS. Diets rich in saturated fat and sugar can induce a low-grade inflammatory response within the mediobasal hypothalamus, especially in the arcuate nucleus, median eminence, and adjacent hypothalamic regions, through microglial activation and the induction of pro-inflammatory pathways such as JNK and NF-κB. This process has been associated with central insulin and leptin resistance, the disruption of energy balance signaling, and altered neuroendocrine regulation. Importantly, hypothalamic inflammatory activity may also affect reproductive control centers: GnRH neurons are anatomically vulnerable to cytokine-mediated signaling, and the repression of GnRH gene expression together with altered LH secretion could contribute to anovulation and menstrual dysfunction. In this framework, hypothalamic inflammation may help explain why obesity, especially when accompanied by PCOS, is associated with heterogeneous reproductive, metabolic, and endocrine disturbances [27] (Figure 1).

Insulin resistance (IR) with secondary hyperinsulinemia is considered a principal mechanism linking obesity to female infertility by altering reproductive endocrine regulation [28]. In addition, obesity-related hyperleptinemia may contribute to anovulation by promoting hyperinsulinemia and exerting direct inhibitory effects on ovarian function; leptin can impair granulosa-cell steroidogenesis and suppress folliculogenesis in a dose-dependent manner [29,30]. Further contributing mechanisms include altered gonadotropin-releasing hormone (GnRH) pulsatility, decreased sex hormone-binding globulin (SHBG), and changes in ovarian/adrenal androgen production and luteinizing hormone (LH) dynamics [9].

Obesity is frequently accompanied by lipotoxicity and a chronic, low-grade inflammatory milieu, which can impair reproductive function, diminish endometrial receptivity, and adversely influence early embryo development, thereby reducing the likelihood of successful implantation and ongoing pregnancy [30,31]. These detrimental reproductive effects appear to be amplified in women with PCOS, a condition with an estimated prevalence of approximately 5–10% in Western populations [30,32]. In a cohort of ovulatory subfertile women, the probability of achieving natural conception within 12 months was reported to decline by approximately 4% for each 1 kg/m^2^ increase in BMI above 29 kg/m^2^ [33,34]. Observational evidence further suggests that bariatric surgery is associated with improvements in ovulation, menstrual cyclicity, and conception rates; current guidance generally recommends postponing pregnancy for 12–18 months after surgery and ensuring careful micronutrient monitoring and supplementation during pregnancy [16]. Additionally, structured lifestyle optimization in conjunction with ART is being evaluated in a randomized controlled trial [8].

### 2.2. Treatment Selection in Obese Infertile Women: Bariatric Surgery vs. GLP-1 Receptor Agonists

In women with obesity and infertility, treatment selection should be individualized and typically follows a stepwise approach that balances reproductive urgency, metabolic risk, and the magnitude/durability of weight loss required. Pharmacotherapy, including GLP-1 receptor agonists (GLP-1RAs), can be considered as an adjunct to lifestyle intervention in adults with BMI ≥ 30 kg/m^2^, or BMI ≥ 27 kg/m^2^ with obesity-related comorbidities, particularly when adequate weight loss is not achieved with lifestyle measures alone [35]. Importantly, GLP-1RAs are not recommended during pregnancy; women of reproductive age should be counseled to use effective contraception during treatment and to discontinue therapy prior to attempting conception. For semaglutide, discontinuation at least 2 months before a planned pregnancy is recommended due to its long half-life [36]. For tirzepatide, pregnancy use is not recommended and the product information advises discontinuation when pregnancy is recognized, with additional counseling regarding reproductive considerations during use [37]. In contrast, MBS is recommended for patients with BMI ≥ 35 kg/m^2^ regardless of comorbidities, and may be considered for BMI 30–34.9 kg/m^2^ with metabolic disease, offering the most durable weight loss and metabolic improvement when medical therapy is insufficient [6]. For women seeking fertility treatment, these options should be discussed alongside timing-to-conception planning, contraception during rapid weight loss, and the need for structured nutritional monitoring, particularly after malabsorptive procedures [6].

### 2.3. Female Obesity, Fertility and Bariatric Surgery (BS)

In women with obesity, BS is supported by clinical guidance as an effective approach for achieving durable weight loss and reducing obesity-related risk [6]. Within reproductive care, BS has been associated with improved fertility and reduced pregnancy-related risk when accompanied by appropriate contraception, micronutrient surveillance, and individualized planning of the interval to conception [16]. Women of reproductive age are estimated to account for approximately 65% of BS recipients annually in the United States. When lifestyle interventions and pharmacologic management do not achieve adequate clinical benefit, BS may improve fertility and mitigate pregnancy-associated comorbidities [14]. Typical postoperative weight reduction exceeds 20% of total body weight, a magnitude associated with improvement in PCOS, type 2 diabetes mellitus, and hypertension [17]. According to international criteria, adults aged 18–60 years with class III obesity (BMI > 40 kg/m^2^) or class II obesity (BMI 35.0–39.0 kg/m^2^) with obesity-related comorbidities may be eligible for BS [38]. The 2022 joint ASMBS/IFSO statement recommends BS for BMI ≥ 35 kg/m^2^ irrespective of comorbidity status and suggests consideration for BMI 30–34.9 kg/m^2^ in the presence of metabolic disease; lower BMI thresholds apply in Asian populations [6]. As a large proportion of women undergoing major BS are between 20 and 44 years of age, perioperative counseling should address contraception, nutritional assessment and supplementation prior to conception, and weight management during and after pregnancy [16]. BS is considered a safe therapeutic option for obesity and is associated with reductions in complications linked to maternal obesity [39].

Bariatric procedures are commonly categorized as restrictive, malabsorptive, or combined restrictive–malabsorptive. Restrictive operations, such as SG, vertical banded gastroplasty (VBG) and its modification silastic ring vertical gastroplasty (SRVG), and adjustable gastric banding (AGB), promote weight loss primarily through reduced gastric volume and consequent limitation of caloric intake. Compared with combined procedures, restrictive operations are often associated with a shorter period of rapid weight loss (approximately 9–12 months versus 12–18 months) [40]. In the United States, combined procedures historically predominated; RYGB accounted for 93% of procedures performed in 2000, increasing from 55% in 1990 [41]. In a randomized study with 10-year follow-up comparing laparoscopic RYGB with laparoscopic adjustable gastric banding (LAGB), laparoscopic RYGB produced greater weight loss (76.2% vs. 46.2%) but was associated with higher complication rates, including serious long-term surgical complications [42]. Currently, SG is the most frequently performed metabolic–bariatric procedure worldwide, comprising more than 60% of total and primary operations [43], and sustained weight reduction is a central outcome after SG [44].

At present, the optimal procedure for improving infertility in women with obesity has not been conclusively established [45]. Nevertheless, BS has been associated with higher conception rates; in some analyses, conception rates increased substantially following marked postoperative weight loss (reported pooled conception improvement of 67%, 95% CI 47–87%, *p* < 0.05), plausibly mediated by favorable changes in reproductive hormones, menstrual cyclicity, and ovulation [46]. In severe obesity, BS remains the most durable weight loss strategy and may restore reproductive function when appropriately selected [6,16]. Additionally, evidence suggests lower risks of gestational diabetes and hypertensive disorders compared with persistently obese controls, with antenatal management tailored to procedure-specific considerations (e.g., alternative approaches to gestational diabetes testing after RYGB and fetal growth surveillance when indicated) [16]. Postoperative weight loss is also associated with improvements in dyslipidemia, blood pressure, glycemia, inflammation, and sleep apnea, which may contribute to reduced long-term cardiovascular morbidity and mortality [19].

#### 2.3.1. Female Fertility and RYGB

##### RYGB Mechanism

RYGB is a combined restrictive–malabsorptive bariatric procedure in which the functional gastric reservoir is reduced to an estimated volume of approximately 15–30 mL [47]. The created gastric pouch is anastomosed to the jejunum after jejunal division roughly 30–75 cm distal to the ligament of Treitz, forming the alimentary (Roux) limb. The excluded biliopancreatic limb, which includes the gastric remnant, is subsequently anastomosed to the small bowel approximately 75–150 cm distal to the gastrojejunostomy [47]. By diverting nutrients away from the proximal small intestine and accelerating delivery to more distal segments, RYGB alters nutrient handling through both reduced intake and malabsorption; the malabsorptive component may attenuate over time as adaptive intestinal hypertrophy occurs [48]. In addition, postoperative changes in enteroendocrine signaling, particularly increased secretion of GLP-1 and PYY, contribute to appetite reduction and improvements in glucose homeostasis [49]. First described by Mason in 1966, RYGB remains, together with sleeve gastrectomy, one of the most widely performed and durable bariatric operations, with consistent long-term outcomes [50,51].

##### Effects of RYGB on Ovarian Reserve and AMH Levels

Multiple investigations have examined postoperative AMH following RYGB, using AMH as a biomarker of ovarian reserve, with inconsistent findings across studies. While several reports describe a reduction in AMH after surgery, fertility improvement after bariatric procedures has also been documented, underscoring uncertainty in the interpretation of AMH changes in this context [52].

In a cohort of 16 women assessed before and after RYGB, AMH decreased by 23.9% among participants aged <35 years, alongside a 14.5% reduction in BMI. In contrast, no significant AMH change was observed in women aged ≥35 years, who had low AMH values both pre- and postoperatively (<1 ng/mL in one older subgroup and <0.1 ng/mL in the oldest subgroup). The authors proposed that the observed AMH decline in younger women might be related to perioperative factors such as surgical stress and/or micronutrient malabsorption potentially influencing AMH-related pathways [52] (Table 1).

A prospective cohort study of 48 women aged 18–35 years with morbid obesity (mean BMI 40.9 ± 3.6 kg/m^2^) evaluated AMH before and after RYGB [53]. AMH rose significantly after a preoperative very low-calorie diet (VLCD) (median 30.0 to 35.0 pmol/L; *p* = 0.014) and then declined at 6 and 12 months after surgery (19.5 and 18.0 pmol/L, respectively; *p* = 0.001) (Table 1). Over the same period, the free androgen index (FAI) decreased markedly at 12 months (1.2 vs. 3.5; *p* < 0.0005), and the AMH reduction exceeded the expected annual age-related decline of 5.6% [54].

In a retrospective analysis of 39 women (18–45 years) undergoing SG or RYGB (23 SG, 16 RYGB; 6 with PCOS), AMH decreased by 18% at 6 months and by 35% at 12 months postoperatively (*p* = 0.010 and *p* = 0.001, respectively), independent of excess weight loss. The authors suggested that postoperative follicular stress might contribute to the reduction and highlighted the need for longer-term data to clarify implications for ovarian reserve [55] (Table 1). A 2023 systematic review including eight studies (*n* = 547) further reported that laparoscopic RYGB is associated with substantial excess weight loss and reductions in testosterone at 6 and 12 months, consistent with improvement in the metabolic and hormonal profile of women with PCOS [56] (Table 1).

AMH trajectories after RYGB remain variably reported, and their relevance to fertility outcomes is not definitively established. Available data indicate that AMH may decline during the first 6–12 postoperative months in some cohorts, whereas other studies report smaller changes or no clear reduction; importantly, AMH patterns do not consistently mirror improvements in clinical reproductive outcomes. Two non-exclusive mechanisms may contribute to this heterogeneity. First, reductions in AMH could reflect altered follicular dynamics during rapid weight loss, potentially influenced by postoperative stress and/or micronutrient status. Second, particularly in PCOS, where AMH is often elevated and correlates with insulin resistance and hyperandrogenism, postoperative AMH reductions may represent metabolic/endocrine normalization rather than clinically meaningful depletion of ovarian reserve. Accordingly, AMH should be interpreted in the context of age, baseline ovarian reserve, reproductive history, and postoperative nutritional status. Until prospective studies relate postoperative AMH trajectories to time to conception and live birth, AMH changes alone should not be used to infer either improved or impaired fertility potential [51,52,53,54,55].

##### Hormonal and Metabolic Improvements After RYGB

RYGB has been associated with substantial improvements in insulin sensitivity and hyperandrogenism among women with PCOS. In the study by Eid GM et al. [57], 24 women with PCOS (mean age 34 years; BMI 50 ± 7.5 kg/m^2^) underwent laparoscopic RYGB, achieving a mean excess weight loss (EWL%) of 56.7 ± 21.2% at 12 months. Regular menstrual cyclicity was re-established in all participants within a mean of 3.4 ± 2.1 months; complete resolution of hirsutism was reported in 52%, and five women conceived spontaneously after surgery, indicating clinically meaningful restoration of reproductive function [57] (Table 1).

Consistent findings were reported by Escobar-Morreale et al. [58] in a cross-sectional study of 36 premenopausal women, 19 of whom had undergone laparoscopic RYGB; 17 participants had PCOS. A marked improvement in insulin resistance was observed, with values decreasing from 5.79 ± 2.78 to 1.6 ± 1.0 (*p* < 0.001), supporting a pronounced metabolic benefit of RYGB in reproductive-aged women [58] (Table 1).

AMH concentrations have been reported to correlate with insulin resistance and androgen status in both PCOS and non-PCOS populations [59]. Lifestyle-induced weight loss alone does not appear to produce consistent changes in AMH [60], whereas hypocaloric dietary interventions combined with sibutramine have been shown to improve hyperandrogenemia and insulin sensitivity while reducing AMH [61]. Accordingly, postoperative reductions in AMH and testosterone after RYGB may be more consistent with improved insulin sensitivity and endocrine normalization than with a decrease in ovarian reserve [62].

##### Fertility and Pregnancy Outcomes After RYGB

Jamal et al. [63] evaluated outcomes in 566 women with morbid obesity who underwent RYGB between 2000 and 2009; 31 patients (5.5%) had PCOS (mean age 32 ± 5.8 years; BMI 52.8 ± 9.08 kg/m^2^). After surgery, 82% reported normalization of menstrual cyclicity, hirsutism resolved in 29%, and complete diabetes remission occurred in 77.8% of those with pre-existing diabetes. Among 10 women who had not conceived prior to surgery, 6 achieved pregnancy within 3 years (5 spontaneous conceptions and 1 via intrauterine insemination), despite recommendations to avoid conception for 18 months postoperatively. The reported 100% conception rate among those previously unable to conceive within 3 years emphasizes the potential fertility benefit of RYGB in women with PCOS [63] (Table 1).

A meta-analysis by Chang et al. [64] reported that bariatric surgery was associated with a higher likelihood of pregnancy compared with metformin therapy alone (34.9% vs. 17.1%). Across 10 included studies (metformin: 5 studies, *n* = 192; bariatric surgery: 5 studies, *n* = 186, comprising 2 RYGB, 2 SG, and 1 combined RYGB + SG), and with longer mean follow-up in the surgical cohorts (metformin 11.2 vs. bariatric surgery 24.5 months), the authors reported increased odds of pregnancy relative to placebo or non-surgical interventions (OR 3.08, 95% CI 1.29–7.37; *p* = 0.01). The pooled pregnancy rate was higher following bariatric surgery than metformin (34.9%, 95% CI 0.20–0.53 vs. 17.1%, 95% CI 0.12–0.23; *p* = 0.026 for between-group difference). Menstrual disorders also improved more markedly in surgical cohorts, with a reported reduction of 92% compared with 54% in the metformin cohorts, although the available data were limited [64] (Table 1).

In addition, a retrospective cohort study with 5-year follow-up reported that both laparoscopic sleeve gastrectomy (LSG) and laparoscopic RYGB (LRYGB) were effective and safe for weight loss and fertility improvement in women with morbid obesity [65]. In that study, 79 women underwent LSG (*n* = 38) or LRYGB (*n* = 41), with evaluation of excess weight loss (%EWL), comorbidity changes, postoperative complications/mortality, and fertility outcomes. Fertility outcomes were assessed across primary infertility (*n* = 21), secondary infertility (*n* = 8), and pre-marriage patients (*n* = 50). No maternal or neonatal complications specifically attributable to diabetes or hypertension were recorded during pregnancy or childbirth [65] (Table 1).

##### RYGB Effects on Long-Term Hormonal and Reproductive Function

A prospective cohort study including 106 women (85 undergoing RYGB and 21 undergoing laparoscopic gastric banding; median BMI 44.5) assessed sexual function and reproductive hormones over a 2-year postoperative period. Participants achieved an approximately 33% reduction from baseline body weight and reported improvements across multiple domains of sexual function, including desire, arousal, lubrication, and satisfaction. Biochemically, total testosterone decreased, SHBG and estradiol increased, and overall reproductive hormone profiles shifted toward improvement after surgery [66] (Table 1).

In contrast, another prospective cohort study of 29 reproductive-aged women undergoing RYGB reported that ovulation frequency (baseline 90%) and markers of ovulation quality did not change materially after surgery. In that cohort, SHBG increased (*p* < 0.001), whereas testosterone and estradiol decreased (*p* = 0.002 and *p* = 0.03, respectively), consistent with endocrine normalization without apparent impairment of ovulatory function [67] (Table 1).

Long-term follow-up data also suggest the durability of weight loss outcomes after RYGB, with 10-year studies reporting maintenance of approximately 20% body weight loss in 73.5% of female patients [15,68]. Nevertheless, if postoperative declines in AMH are subsequently shown to represent true compromise of ovarian reserve, consideration of fertility preservation counseling prior to bariatric surgery may be appropriate for reproductive-aged women [55].

#### 2.3.2. Female Fertility and VSG

##### VSG Mechanism

SG involves the removal of the greater curvature of the stomach, reducing its volume by 75–85% [69,70], thereby limiting food intake. This procedure also removes ghrelin-secreting endocrine cells located in the greater curvature of the stomach, thereby helping to reduce appetite [69]. Weight loss, together with changes in other metabolic hormones, improves glucose homeostasis and contributes to better glycemic regulation [49].

SG is the most common bariatric metabolic procedure [6], accounting for more than 60% of both total and primary procedures globally [43]. Effective and durable weight loss is the principal outcome after SG [44]. This treatment is also chosen because of its well-established safety record, effectiveness, and noticeably shorter postoperative recovery period [6].

##### VSG Effects on PCOS and Menstruation

1. VSG Effects on Restoration of Menstrual Cycles and Infertility

In the prospective multicenter cohort study “Sleeve Gastrectomy for Obese Polycystic Ovary Syndrome” (SGOP), designed to evaluate SG efficacy in women with obesity and PCOS, 79.03% (181/229) achieved remission of menstrual disorder at 12 months after SG, with mean percent total weight loss (TWL%) of 33.25 ± 0.46% [44]. In the same cohort, 60.7% (139/229) attained regular menstrual cycles during the early postoperative period (within the first to third month after SG). The study included 229 women with obesity and oligo/anovulatory PCOS (mean age 28.68 years; mean BMI 40.91 kg/m^2^) followed for 1 year; remission of irregular menstruation was defined as a spontaneous, consecutive 6-month cycle pattern with cycle length 21–35 days within the first postoperative year [44].

**Table 1:** Clinical, hormonal and fertility outcomes of RYGB bariatric surgery in women with obesity.

Ref. No	Sample Size	Age (Years)	Time Point	BMI (kg/m^2^)	Weight Loss (kg)	Parameter	Surgical Procedure	Fertility Outcome	Country	Follow-Up
Eid et al. (2005) [57] Retrospective cohort	24 PCOS women	34 ± 9.7 y	Pre-op 12–57 months	50 ± 7.5	56.7 ± 21.2%.	Hirsutism, irregular menstrual cycles, infertility	LRYGB	5 infertile pre-op conceive no aid of clomiphene. 75% complete resolution of hirsutism improvement with type II diabetes mellitus, hypertension, and dyslipidemia. 1 y mean post-op BMI 30 ± 4.5 Menstrual dysfunction pre-op 24 PCOS 100% change post-op	USA	27.5 ± 16.5 months
Escobar-Morreale et al. (2005) [58] cross-sectional study	36 premen/sal obese women (17 obese PCOS women)	27 ± 1.5 y (obese PCOS group)	Pre-op 6 months post-op	37.1 ± 1.5	41 ± 9 kg	Hyperandrogenism, (hirsutism score 9.5 ± 6.8 to 4.9 ± 4.2) insulin resistance 5.79 ± 2.78 to 1.6 ± 1.0, menstrual function.	LRYGB (19 women)	restoration of regular menstrual cycles, ovulation in all patients, improvement in insulin resistance hyperandrogenism	Spain	6 months 12 ± 5 months
Mehri et al. (2008) [52] Prosperity cohort study	16 obese women	Group 1: 29.5 ± 1.0 y (*n* = 7) and Group 2: 40.5 ± 2.4 y (*n* = 4) and Group 3: 51.2 ± 2.8 y (*n* = 5)	Pre-op and mean 87 ± 30 days post-op	Group 1: Pre 50.2, Post 42.9 Group 2: Pre 44.4, Post 35.8 Group 3: Pre 48.7, Post 42.9	Not directly reported (14.5–19.4% drop)	AMH Group 1: Pre:3.8 and Post:2.9, Group 2: Pre:0.63 and Post:0.80, Group 3: Pre:0.10 and Post:0.10 History of PCOS: Group 1: Yes 1, No 6, Group 2: Yes 1, No 3, Group 3: Yes 0, No 5	RYGB LAGB	AMH ↓ 23.9% in women < 35 y post-op no change in older groups; no direct fertility data; implications on ovarian reserve	USA	~3 months post-op
Jamal et al. (2011) [63] Retrospective cohort with interviews	566 morbidly obese women	32 ± 5.8 y	Baseline	52.8 ± 9.08 kg/m^2^ (range 37–76) pre-op	mean %EWL 64%, (26% in the first month post-operative)	31 (5.5%) PCOS 50% of the patients with PCOS infertile, 85% menstrual dysfunction, 70% had hirsutism, 45% T2DM, 40% depression	RYGB	menstruation corrected in 82%, hirsutism resolved in 29%, 77.8% of those with diabetes had complete remission, 100% conception rate within 3 y of surgery	USA	46.7 ± 35.3 months
Legro et al. (2012) [67] Prospective cohort	29 obese women	18–40 y	Baseline 1 m, 3 m, 6 m, 12 m, 24 m	49 −6 −11 −15 −18 −19	−15 −29 −40 −50 −51	ovulation frequency/quality Ovarian volume (BL) 16.1 ± 13.1 (6 m) 17.2 ± 9.4 (12 m) 13.3 ± 6.4 MCL(d) 37.2 −2.5 −2.9 −6.0 −4.4 −4.1 FPL(d) 22.0 −3.2 −6.5 −8.2 −7.9 −8.9 LPL(d) 12.2 3.8 0.0 0.8 0.8 0.0	RYGB	Ovulatory cycles %, BL 90%, 1 m 9.9%, 3 m 10%, 6 m 10.1%, 12 m 10%, 24 m 0.98% SHBG ↑ TESTO ↓ E2 ↓	USA	24 months post-operative
Sarwer et al. (2014) [66] Prospective cohort	106 morbidly obese women	34–48 y	Model based estimates, mean Baseline: 1 y, 2 ys model based mean changes (95%CI): y 1 from BL, y 2 from BL, y 2 from y 1	44.5	32.7% (95% CI, 30.7–34.7%) (1 y), 33.5% (95% CI, 31.5–35.6%) (2 ys)	E2 pg/mL 86.9/51.4/53.1 −35.5/−33.8/1.7 total testo ng/dL: 47.8/30.4/23.1/−17.4/−24.7/−7.3 FSH mIU/mL: 15.3/22/29.9/6.7/14.6/7.9 LH mIU/mL: 9.4/13.3/15.9/3.9/6.5/2.6 SHBG μg/mL: 4.8/11.4/9.8/6.6/4.9/−1.6 DHEAs μg/mL: 118.6/106.1/92.6/−12.4/−26.0/−13.6	LRYGB (85) 80.2% AGB (21) 19.8%	improvements in sexual functioning, reproductive hormone levels,	USA	2 y
Nilsson-Condori et al. (2018) [53] Prospective cohort	48 morbidly obese women	18–35 y	Baseline, pre-surgery (post-diet), 6 months, 12 months post-operative	40.9 (38.6 after VLCD) → 25.4 (6.4) (post-operative)	After VLCD 110.3 ~41.7, 75.5 (after 12 months)	AMH and FAI/AMH 30.0 (Baseline) 35.0 (after VLCD) 19.5 (6 m), 18.0 (12 m) Testosterone 1.1 after VLCD, 1.0 after 6 m, 0.9 after 12 m LH 6.1, 5.9 after VLCD, 5.9 after 6 m, 5.3 after 12 m FSH 5.5, 4.8 after VLCD, 4.2 after 6 m, 4.3 after 12 m SHBG 28.0, 39.5 after VLCD, 67.0 after 6 m, 73.0 after 12 m	RYGB	↓ AMH; ↓ FAI (suggesting improved fertility). 2 y after improvements in overall sexual functioning (arousal, lubrication, desires, and satisfaction).	Sweden	12 months (post-operative)
Vincentelli et al. (2018) [55] Prospective cohort	39 obese women, 6 PCOS, 79% morbid obesity	18–45 y	Baseline 6 months 12 months	45.4 33.6 31.4	61.7 70.2	AMH 13.4 11.2 10.5	LRYGB (16) SG (23)	↓ in AMH negative impact of BS on ovarian reserve	France	12 months (post-operative)
Chang et al. (2020) [64] Retrospective cohort survey—meta-analysis	Metformin: 5 studies, *n* = 192; BS: 5 studies *n* = 186	33–40 y	22 months post-operative	Pre-op: 44.5 (mean)	27.3% (mean EWL)	Pregnancy and infertility history questionnaire	RYGB (2) SG (2) RYGB + SG (1)	Pregnancy rate 13.3% to 53.6% post-operative; infertility, 18.8% to 5.4%. pregnancy rate BS vs. metformin, 34.9% vs. 17.1%. Improvement in menstrual disorders BS vs. metformin ↓ 92% vs. ↓ 54%	Taiwan	22 months post-operative (mean)
Ghobrial et al. (2023) [56] Prospective cohort	547 obese PCOS women	30–41 y	Pre-op 6 and 12 months post-operative	Pre-op mean: 43.7 ± 6.1	EWL in 8 studies	Serum AMH, hormonal profile, ovulation	Laparoscopic RYGB	Significant ↑ in AMH, regular menstruation resumed, ovulation improved, ↓ TESTO	Switzerland	6 and 12 months post-operative
Shehata et al. (2025) [65] Prospective cohort	79 morbidly obese women	LSG: 27.4 ± 5.5 (18–37 y) RYGB: 26.6 ± 5.7 (19–38 y)	5 y	LSG: 43.2 ± 8.2 RYGB: 46.1 ± 11.4	EWL% LSG: 63.1 ± 1.5%, RYGB: 65.2 ± 8.1%, 5th y %EWL: 50–103%	Pregnancy conception rates %EWL	LSG: 38 RYGB:41	High conception rates post-operative (primary infertility: LSG 80%, RYGB 72.7%; secondary: 100% both; pre-marriage: LSG 100%, RYGB 92.3%), min pregnancy complications, no neonatal anomalies	Egypt	5 y

Notes: PCOS—polycystic ovary syndrome; EWL—excess weight loss; SG—sleeve gastrectomy group; LMT—lifestyle modification therapy group; MCL—menstrual cycle length (d); FPL—follicular phase length (d); LPL—luteal phase length (d); VLCD—very low-calory diet; y—year.

The authors concluded that SG is associated with clinically significant improvement in menstrual regularity in this population and that TWL% may be a more informative predictor of menstrual remission than endpoint BMI (Table 2) [44].

Comparable findings have been reported in additional cohorts, including a Chinese single-center study with a remission rate of approximately 78%, whereas an Iranian study reported a lower rate of 66% [71,72]. Another Chinese study enrolling 88 women with obesity and PCOS and 76 control patients aged 18–45 years (recruited between May 2013 and December 2020) reported mean percent excess weight loss (%EWL) and percent total weight loss (%TWL) at final follow-up of 97.52% ± 33.90% and 31.65% ± 10.31%, respectively. In that cohort, the proportion of women reporting monthly menstrual cycles increased significantly by 6 months (75.86%) and at final follow-up (79.52%) after LSG (*p* < 0.05). In this study, menstrual restoration required a consecutive 3-month cycle pattern [73] (Table 2). Menstrual irregularity and prolonged cycle length have also been associated with increased risks of type 2 diabetes mellitus (T2DM), gestational diabetes mellitus (GDM), and coronary heart disease [74,75].

A cross-sectional study including 387 Saudi women who had undergone SG between December 2023 and May 2024 reported that 70.5% of participants experienced postoperative changes in their menstrual cycle, consistent with an effect of weight loss and metabolic improvement on menstrual characteristics [76]. Although regional variation was observed (western region 78.8% vs. central region 59.5%), no statistically significant association between region and postoperative menstrual cycle change was identified (*p* = 0.140) [76]. However, the sample was limited to Saudi Arabian tribal populations.

In a separate cross-sectional survey of 515 premenopausal women (70.3% SG; 29.7% RYGB), the proportion reporting irregular menstruation decreased from 38.6% preoperatively to 25.0% postoperatively. Mean excess weight loss (EWL) was 74.0 ± 30.4%, and 82.0% achieved ≥50% EWL [77] (Table 2). Alhumaidan et al. [78] similarly reported a reduction in menstrual irregularities from 41.9% to 36.2% in a cohort of 516 Saudi women undergoing various bariatric procedures (85.9% SG) with mean weight loss of 54.2 kg; postoperative menstrual cycle regularity was reported in 26.3%, and 4.9% reported pregnancy after surgery [78] (Table 2). Additionally, another study reported complete resolution of amenorrhea among premenopausal women after SG; in that cohort (procedures performed May 2007–December 2011), 117 patients (87 women) were evaluated over 2 years (baseline mean weight 135.6 ± 23.7 kg; mean BMI 46.6 ± 6.0 kg/m^2^; mean age 40.3 ± 10.7 years). At 24-month follow-up, 2 of 12 women categorized as infertile preoperatively had delivered two healthy children [79] (Table 2).

2. Hormonal Changes following VSG: AMH, Androgen and Ovarian Reserve

Regarding AMH, a prospective study reported an overall increase following SG. In that cohort of 53 women with obesity (mean age 32.4 years; mean BMI 44.8 kg/m^2^) evaluated at 3 and 6 months after SG, AMH demonstrated a progressive rise postoperatively [80]. Mean AMH values increased to 3.4 ng/mL at 3 months and 4.8 ng/mL at 6 months; the difference between baseline and 6 months was statistically significant (*p* < 0.005), whereas the change between baseline and 3 months was not significant (Table 2) [80].

Conversely, other data suggest that AMH may show a transient postoperative decline after VSG, potentially reflecting metabolic changes rather than a reduction in ovarian reserve [81]. A cohort study of 75 women with obesity (43 with PCOS and 32 without PCOS), aged 20–35 years with mean BMI 43.95 kg/m^2^, evaluated AMH in relation to weight change following SG [82]. Preoperatively, AMH was lower in the non-PCOS group than in the PCOS group (mean AMH 1.77; mean BMI 45.03; mean age 29.34 vs. mean AMH 4.68; mean BMI 42.52; mean age 27.77, respectively). At 6 months after SG, BMI decreased significantly and AMH was reduced in both groups (non-PCOS: BMI 32.67 ± 3.51; AMH 1.18 ± 0.84; PCOS: BMI 30.76 ± 2.93; AMH 3.38 ± 1.21) (Table 2) [82].

Wang et al. [83] assessed SG in 24 women with obesity and PCOS (mean weight 99.8 kg) and compared outcomes with 24 women with obesity and PCOS (mean weight 89.7 kg) managed with lifestyle modification therapy (LMT), including dietary, exercise, and behavioral interventions. SG was associated with a significant reduction in androgen concentrations (0.562 ± 0.07 to 0.31 ± 0.10 ng/mL; *p* = 0.012), whereas androgen changes in the LMT group were not significant (*p* > 0.05). Weight reduction was greater after SG (15.7 kg at 3 months; *p* < 0.0001) than with LMT (4.1 kg at 3 months and 5.7 kg at 6 months; *p* < 0.05). Restoration of cyclicity and ovulation occurred in 20/24 (70.8–83.3%) participants after SG within 3–6 months, compared with 6/24 (25%) in the LMT group at 3 months. Collectively, SG was associated with greater weight loss and more favorable endocrine and reproductive outcomes than LMT in women with obesity and PCOS (Table 2) [83].

##### Weight Loss and Fertility Outcomes After VSG

Although fertility rates may increase after BS in women with obesity, conception within the first 18 months postoperatively is generally discouraged, primarily due to concerns regarding maternal–fetal risk during the phase of rapid weight loss and potential nutritional instability. In this context, achieving weight stabilization prior to pregnancy after SG is considered important, with the goal of optimizing endocrine regulation and improving pregnancy and live birth outcomes [84,85].

In a 2018 retrospective study, the presence of PCOS did not adversely affect weight loss following SG. In that analysis, 119 women with PCOS were compared with 119 women without PCOS who underwent SG between 2008 and 2016 (overall mean age 35.5 ± 10.7 years; mean preoperative BMI 41.9 ± 5.2 kg/m^2^). At 12 months, %EWL was higher in the PCOS group than in controls (66% vs. 60%; *p* = 0.05). Follow-up attendance and weight loss trajectories were reported as follows: 222 patients (93%) attended the 3-month visit with mean ΔBMI −6.8 kg/m^2^ and 38.1 ± 11.6% EWL; 206 patients (89%) attended at 6 months with mean ΔBMI −10.1 kg/m^2^ and 52.9 ± 15.2% EWL; and 200 patients (84%) attended at 12 months with mean ΔBMI −12.1 kg/m^2^ and 62.9 ± 21.1% EWL. Within the PCOS subgroup, mean weight loss was 55.4% at 6 months and 65.8% at 12 months. The authors also reported that 22% of women with PCOS conceived within 12 months after surgery (69% previously nulliparous), suggesting a potential fertility benefit following SG in this population [85] (Table 2).

A retrospective series from Adıyaman Medical Faculty Training and Research Hospital (Turkey; May 2014–December 2019) described outcomes in 23 women with morbid obesity (BMI > 40 kg/m^2^) who underwent LSG. PCOS was present in 15/23 (65.2%); with one exception, all women with PCOS reportedly achieved childbirth after surgery. BMI declined significantly after LSG compared with preoperative values (*p* = 0.00001), and the postoperative conception and live birth rates were 91.3% and 65.2%, respectively [86] (Table 2).

More broadly, Kort et al. [87] reported that reproductive outcomes were substantially better among women achieving ≥10% weight loss compared with <10% weight loss. Specifically, pregnancy rates were 88% versus 54%, live birth rates 71% versus 37%, and spontaneous conception rates 35% versus 17%, respectively, indicating the importance of adequate weight reduction in optimizing reproductive outcomes after BS [87].

Musella et al. [88] reported fertility and live birth outcomes among infertile women with obesity, noting an overall fertility rate and live birth rate of 62.7% (69 pregnancies among 110 women who had previously been unable to conceive prior to weight loss). Although higher pregnancy rates were observed among women undergoing SG, the authors did not identify differences in fertilization rates across procedures and concluded that baseline BMI and the magnitude of postoperative weight loss were key determinants of achieving pregnancy (Table 2) [88].

**Table 2:** Clinical, hormonal and fertility outcomes of VSG bariatric surgery in women with obesity.

Ref. No	Sample Size	Age (Years)	Time Point	BMI (kg/m^2^)	Weight Loss (kg)	Parameter	Surgical Procedure	Fertility Outcome	Country	Follow-Up
Musella et al. (2012) [88] Retrospective study	110 infertile obese women	~29 y (mean)	≥2.5 ys	~44 (pre-op), 34.9 ± 2.1 kg/m^2^ after SG, 35.4 ± 0.5 kg/m^2^ gastric bypass, 34.3 ± 2.3 kg/m^2^ after adjustable gastric	Not reported	Weight loss post-surgical BMI as pregnancy predictors	Sleeve gastrectomy, gastric bypass, adjustable gastric banding	62.7% became pregnant; all led to live births	Italy	≥2.5 ys
Vage et al. (2014) [79] Prospective cohort study	117 patients (87 morbid obese women)	40.3 ± 10.7 y (mean)	Pre-op vs. 24 months post-operative	46.6 ± 6.0 (baseline), 12 months: 30.3 ± 5.9, 24 months: 30.6 ± 5.6	~45 kg	Obesity comorbidities (incl. amenorrhea)	LSG	100% resolution of amenorrhea (resumed menses), amenorrhea: Pre-op 17.1%, 12 months 4.6%, 24 months 0% Infertility: Pre-op 17.1, 24 months 7.7%	Norway	24 months
Wang et al. (2015) [83]	24 (SG) vs. 24 (LMT) All PCOS	25.5 y (22–35 y range)	6 months	35.2 (29–45.7 range) Pre-op: 35.2 ± 6.2 1 month post-operative: 32.6 ± 6.7 (reduction 2.7 ± 0.7, EWL 0.43 ± 0.48), 3 months post-operative: 29.9 ± 6.5 (reduction 5.3 ± 1.5, EWL 0.81 ± 0.85), 6 months post-operative: 27.8 ± 4.9 (reduction 7.8 ± 2.9, EWL 1.02 ± 0.92)	22.3 kg (at 6 months post-SG), pre-op: 99.8 ± 22.4, 1 month post-operative: 92.4 ± 23.0 3 months post-operative: 84.1 ± 21.8 6 months post-operative: 77.8 ± 16.8	Menstrual cycle and ovulation recovery	SG (vs lifestyle)	83% regained normal menses and ovulation (SG) vs. 25% with lifestyle. ↓ androgen levels from 0.562–0.07 ng/mL pre-op to 0.31–0.1 ng/mL post-operative. improvement in PCOS symptoms	China	6 months
Bhandari et al. (2016) [81] Cohort study	75 (43 PCOS, 32 non-PCOS)	20–35 y (range), mean PCOS group mean 27.77 y non-PCOS group 29.34 ys	6 months	43.95 kg/m^2^ (mean pre-op) Pre-SG mean BMI: 42.52, Non-PCOS mean BMI 45.03	Not reported (≈55% EWL at 6 months)	AMH level	SG	Menstrual function normalized; AMH levels ↓ Pre-SG mean AMH 4.68, non-PCOS mean AMH 1.77, after 6 months ↓ BMI and AMH levels in both groups, non-PCOS group BMI: 32.67 ± 3.51, AMH: 1.18 ± 0.84, PCOS group BMI: 30.76 ± 2.93, AMH: 3.38 ± 1.21	India	6 months
Dilday et al. (2019) [85] Retrospective study	119 obese PCOS patients, 119 obese non-PCOS patients	35.5 ± 10.7 y	3 months, 6 months, 12 months	Pre-op BMI 42.2 PCOS Pre-op BMI 41.5 non-PCOS	Mean weight loss in PCOS 55.4% (6 months), 65.8% (12 months) and 66% EWL after 12 months non-PCOS patients (60%), 3 months: 93% patients −6.8 kg/m^2^ and 38.1 ± 11.6% EWL, 6 months: 89% patients −10.1 kg/m^2^ and 52.9 ± 15.2% EWL, 12 months: 84% patients −12.1 kg/m^2^ and 62.9 ± 21.1% EWL	BMI %EWL (weight loss)	SG	22% PCOS patients pregnant within 12 months	USA	12 months
Pilone et al. (2019) [80] Prospective cohort study	53 obese women	32.4 y	3 months 6 months	44.8 kg/m^2^ (pre-op mean)	Not reported 29.5% EWL at 6 months	AMH	(LSG)	↑ AMH. Menstrual cycles regularized; dysmenorrhea resolved by 6 months.	Italy	3 and 6 months
Różańska-Walędziak et al. (2020) [77] Cross-sectional study	515 obese women (post-bariatric survey)	37.4 ± 7.7 y (at survey)	1 pre-op y vs. ~3 ys post-operative	42.2 ± 7.5 (baseline) Current BMI 29.8 ± 6.3 BMI loss 12.4 ± 6.3	35.3 ± 17.9 kg (at 2 years); EWL 74.0 ± 30.4%; 82.0% of the patients achieved 50% EWL	Menstrual irregularity and hormones	LSG (70.3%), RYGB (29.7%)	Irregular cycles 38.6% pre-op → 25.0% after surgery (improved regularity)	Poland	Median 37.4 months
Hu et al. (2022) [72] Prospective non randomized trial	90 obese women (81 completed; 41 SG vs. 40 med)	~28 (range 18–40)	Pre-op vs. 12 months post-operative	BMI ≥ 27.5 kg/m^2^ Median BMI at endpoint 30.1 kg/m^2^ in the drug group and 23.7 kg/m^2^ in the surgical group	~34 kg (mean loss)	PCOS remission (cycles + pregnancy)	Sleeve gastrectomy (LSG)	78% PCOS complete remission after SG (vs. 15% with meds) Nearly 95% endpoint BMI below the cutoff values achieved complete remission.	China	12 months
Cai et al. (2023) [73] Prospective study	88 PCOS vs. 76 control obese women	18–45 ys (28.7 ys median)	Pre-op vs. 6 months post-operative	37.4 (median baseline PCOS)	Not reported EWL 97.52 ± 33.90% TWL 31.65 ± 10.31%	Menstrual recovery (regular cycles) Increased risk of T2DM, GDM	LSG	75.86% regained regular cycles by 6 months (from ~0% pre) Final follow-up 79.52% after LSG	China	Mean 3.23 ys
Öner et al. (2023) [86] Retrospective analysis	23 morbidly obese women	31.3 ± 5.1 y (mean)	12 months (1 year)	45.04 ± 3.43 (pre-op mean) 28.65 ± 3.14 (12 months post-operative)	Not reported	LH, FSH, PRL, E2, TESTO levels, time to pregnancy	LSG	91.3% conceived; 71.42% gave birth after LSG, 28.58% aborted, 65.21% live birth rate, 82.6% women in IVF prior to LSG, 57.89% had children after LSG, 66.67% natural conception after surgery, 13.33% both NC and IVF, 20% following IVF only	Turkey	5 ys
Alamdari et al. (2024) [71] Single-center study	50 obese PCOS women	31.69 ± 9.54 y	A y post-operative	Mean BMI before surgery 44.28 ± 3.03 kg/m^2^, mean BMI after surgery 29.37 ± 2.41 kg/m^2^		PCOS (Clinical signs, symptoms, hormonal assessments)	VSG	Oligomenorrhea improved 66% (of patients.), PCOS improved 74% (of patients), mean FSH, testo, DHEAs improved (in all patients), ↓ LH, ↓ LH/FSH ratio, ↓ estrogen noted in patients with improved clinical response	Iran	1 y
Alhumaidan et al. (2024) [78] Online survey	516 obese women	18–50 y (37.2% age 18–30 y)	Pre- vs. post-surgery (self-reported)	Not reported	54.2 kg (mean lost)	PCOS (12.4%), hormonal imbalances (2.5%), menstrual abnormalities, co-morbidities (1.6%)	85.9% SG + various BS	Menstrual irregularities from 41.9% to 36.2%, menstrual cycle regularity 26.3%, 4.9% pregnant, no change in cycle frequency (10.6 cycles/yr); slight decrease in flow/duration	Saudi Arabia	Varied (~0–2 ys post-operative)
Alsareii et al. (2024) [76] Cross-sectional study	387 post-LSG women	~34 y (18–55 y range)	Post-surgery survey (varied times)	Not reported	Not reported (87% lost weight)	Menstrual changes (cycle regularity)	SG	70.5% (*n* = 273) menstrual changes, 26.3% (*n* = 102), regular cycles (post-operative) vs. 5% pre-op; 4.9% pregnant.	Saudi Arabia	1 to 12+ months post-operative
Zhao et al. (2024) [44] Prospective multicenter cohort study	229 women with obesity + PCOS	28.68 ± 0.4 (mean)	Pre-op vs. 1 y post-operative	40.91 kg/m^2^ (mean BMI)	Not reported TWL 33.25 ± 0.46% TWL% 1 month 12.27 ± 0.21% TWL% 3 months 21.90 ± 0.33% TWL% 6 months 29.46 ± 0.40% TWL% 12 months 33.25 ± 0.46%	Menstrual irregularity (PCOS)	SG	79.03% regained regular menstruation, 21–35 days menstrual cycle after SG, 31.0% regular menstrual cycles in the first month after SG, 60.7% achieved regular menstrual cycles the third month after SG	China	1 year

Notes: PCOS—polycystic ovary syndrome; EWL—excess weight loss; LSG—laparoscopic sleeve gastrectomy; SG—sleeve gastrectomy group; LMT—lifestyle modification therapy group. All fertility outcomes refer to post-surgery results in the respective study populations, AMH—anti-Müllerian hormone; y—year.

### 2.4. Molecular Mechanisms Known So Far to Be Involved in Female Fertility Improvement After Weight Loss Following Bariatric Surgery (RYGB, VSG)

**Figure 2:**
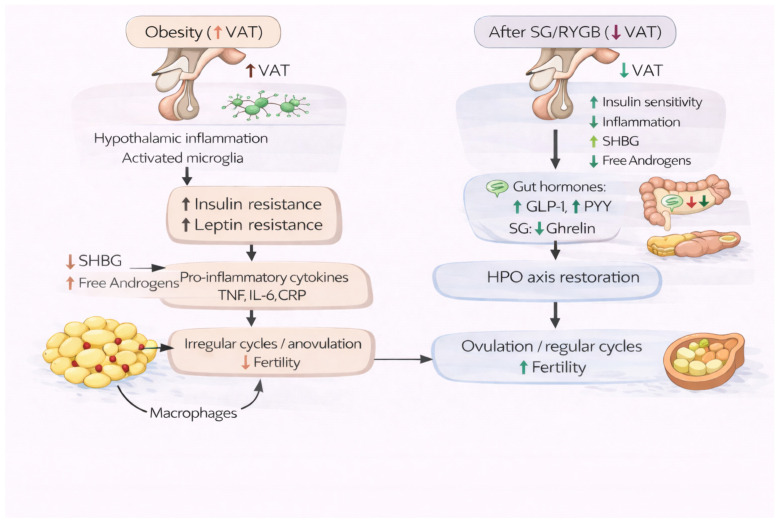
Obesity-related disruption of the hypothalamic–pituitary–ovarian (HPO) axis and its reversal following sleeve gastrectomy (SG)/Roux-en-Y gastric bypass (RYGB). Cartoon image illustrates alterations associated with excess visceral adipose tissue (VAT) and their post-surgical normalization, as described in the main text.

To improve readability, the key mechanistic pathways linking obesity to HPO axis dysfunction and subfertility, as well as their reversal after SG or RYGB, are summarized in Figure 2. In brief, obesity-related visceral adiposity promotes insulin resistance/hyperinsulinemia, leptin dysregulation, chronic low-grade inflammation, and reduced SHBG with increased free androgen availability, collectively impairing GnRH-gonadotropin signaling and ovulatory function. Following SG/RYGB, visceral fat reduction and metabolic improvement, together with gut hormone changes (notably increased GLP-1 and PYY, and procedure-specific effects on ghrelin), contribute to decreased inflammation and androgen excess and support restoration of HPO axis function, menstrual cyclicity, and ovulation (Figure 2).

#### 2.4.1. Visceral Adipose Tissue (VAT) Loss and Total Weight Loss (TWL) After SG and RYGB

Studies demonstrate that both SG and RYGB induce marked VAT loss within the first few postoperative months, with reductions ranging from 35% to 55% after 6–12 months. Total weight loss (TWL) generally ranges from approximately 20% to 35%, depending on procedure and follow-up duration. Whole-body magnetic resonance imaging (MRI) provides accurate evaluation of the effects of these surgical procedures on individual tissue composition [88,89,90,91].

#### 2.4.2. Endocrine Changes, AMH, and HPO Axis Normalization

The magnitude of surgery-induced weight reduction and the accompanying decrease in VAT are key drivers of endocrine changes in women with obesity and are associated with improved menstrual cyclicity, ovulatory function, and reproductive outcomes, in addition to metabolic benefits [14]. Postoperative AMH findings are inconsistent: some studies describe decreases in AMH, whereas others report stable values or findings interpreted as preserved/improved functional ovarian reserve. Clinically, AMH is widely regarded as the most informative hormonal biomarker of ovarian reserve, reflecting the pool of available follicles at a given time point and providing prognostic information regarding ovarian responsiveness during IVF treatment [52,55,56,80,81]. Importantly, a postoperative decline in AMH should not be interpreted as synonymous with oocyte depletion. Rather, particularly in women with PCOS, lower AMH after weight loss may reflect improvement in hyperandrogenism and insulin resistance, with reduced recruitment and accumulation of small antral follicles, a typical PCOS feature, rather than a true reduction in ovarian reserve [92,93]. Moreover, improvement in insulin resistance and androgen excess is associated with normalization of LH secretion and restoration of a more physiologic LH/FSH balance, changes that are closely linked to improved ovulatory function [94]. By reducing insulin-mediated potentiation of LH-driven ovarian androgen production and by improving hypothalamic GnRH pulsatility, these endocrine adaptations support the re-establishment of coordinated gonadotropin release [94,95].

#### 2.4.3. SHBG Increase and Androgen Reduction After Bariatric Surgery

Reduction in VAT is associated with increased concentrations of sex hormone-binding globulin (SHBG), likely through the attenuation of insulin-mediated inhibition of hepatic SHBG production. This rise in SHBG decreases circulating free testosterone and overall free androgen bioavailability; accordingly, while total testosterone may decrease or remain unchanged, free testosterone typically declines, consistent with improved endocrine balance after weight loss [96,97]. In addition, BS has been linked to reductions in both adrenal- and ovarian-derived androgens, including dehydroepiandrosterone sulfate (DHEAS) and androstenedione, with effects that may be particularly evident in women with PCOS. These androgen reductions are thought to be mediated by improved insulin sensitivity, concurrent improvements in clinical hyperandrogenism (e.g., hirsutism), and decreased obesity-related inflammatory activity [96,97].

#### 2.4.4. Inflammation and Biomarkers After Bariatric Surgery (CRP, TNF, IL-6)

Low-grade chronic inflammation in adipose tissue contributes to the circulation of inflammatory markers, and for this reason, BS follow-up studies frequently measure markers such as C-Reactive Protein (CRP), Tumor Necrosis Factor (TNF) and/or Interleukin 6 (IL-6). IL-6 is mostly reported to decrease after the surgical procedure. CRP originates primarily from the liver. Still, it is considered a marker of adipose inflammation as the liver is highly affected by obesity and CRP has consistently been shown to be upregulated with obesity. After BS, CRP levels show rapid declines that persist for up to 10 years, postoperatively [89].

#### 2.4.5. Adipokines (Leptin, Adiponectin) and Restoration of HPO Axis Function

Adipose tissue exerts important endocrine effects through the systemic release of adipokines. Key adipokines include leptin, which signals energy sufficiency, contributes to appetite regulation in states of increased fat mass, and promotes pro-inflammatory immune activity, and adiponectin (APN), which acts on peripheral tissues (including liver and skeletal muscle) to enhance insulin sensitivity, increase fatty acid oxidation, regulate lipoprotein metabolism, and suppress hepatic gluconeogenesis, thereby exerting anti-inflammatory and metabolic effects [98]. Following SG and/or RYGB, the marked reduction in VAT is associated with decreased circulating leptin concentrations, consistent with improved leptin sensitivity and potential facilitation of HPO axis recovery. In parallel, increases in adiponectin have been reported, with some data suggesting a more pronounced rise after RYGB compared with SG. These differential adipokine responses after RYGB may relate to procedure-specific intestinal hormone changes that influence weight loss and downstream metabolic adaptations [98,99]. In women with PCOS, postoperative weight loss has also been associated with reductions in CRP and increases in adiponectin, although improvements may occur more gradually than in women with obesity without PCOS, potentially reflecting underlying predisposition to insulin resistance [100,101].

#### 2.4.6. Gastrointestinal Hormones (PYY, GLP-1, Ghrelin) and the Gut–Brain Axis

In addition to adipokine-mediated effects, both RYGB and SG produce clinically relevant alterations in gastrointestinal hormone secretion that modulate appetite regulation, glucose handling, and insulin sensitivity, thereby contributing to the metabolic–endocrine milieu that supports reproductive axis function. Numerous gut-derived peptides signal to peripheral organs and the central nervous system to regulate glucose homeostasis. In the present context, key hormones include peptide YY (PYY), glucagon-like peptide-1 (GLP-1), and ghrelin. SG is typically associated with sustained suppression of ghrelin as a consequence of gastric fundus resection, whereas ghrelin concentrations after RYGB more commonly return toward baseline or may increase. In contrast, both SG and RYGB elicit marked postprandial increases in GLP-1 and PYY, largely attributable to more rapid nutrient delivery to distal intestinal segments compared with preoperative physiology or diet-induced weight loss [102,103,104,105,106]. Ghrelin is a 28-amino acid gastric peptide involved in hunger signaling that stimulates growth hormone release and is associated with reduced insulin secretion. GLP-1 and PYY are secreted predominantly by distal intestinal L cells; both promote satiety and slow gastrointestinal transit, with PYY improving insulin sensitivity and GLP-1 acting as an incretin that augments glucose-stimulated insulin secretion [103].

#### 2.4.7. Integrated Pathway: From Weight Loss to Improved Fertility Outcomes

The endocrine adaptations observed after bariatric surgery contribute to sustained reductions in adiposity and insulin resistance, thereby supporting improvement in female reproductive function. As adipose tissue decreases, obesity-associated endocrine disturbances, including chronic low-grade inflammation, leptin resistance, and hyperinsulinemia, which negatively influence HPO axis function, tend to improve; circulating leptin concentrations decline toward physiologic ranges and inflammatory markers are reduced, changes that are consistent with partial restoration of HPO axis activity [98,99]. Procedure-related suppression of ghrelin, together with the enhanced postprandial secretion of GLP-1 and PYY, modulates hypothalamic appetite-regulatory pathways by increasing satiety, reducing energy intake, and improving glucose-dependent insulin action, thereby facilitating negative energy balance and visceral fat loss; increased PYY may further contribute to improved insulin sensitivity [102]. Although GLP-1 and PYY exert robust effects on central appetite regulation and energy homeostasis, current human evidence does not confirm a direct effect on GnRH neurons or GnRH pulsatility, and this remains an area of active investigation. Experimental data, including rodent studies, suggest that gut hormones may influence reproductive hormone secretion; however, in humans, effects on reproductive function likely reflect a combination of direct receptor-mediated pathways and indirect mechanisms mediated by weight loss and metabolic improvement [107]. In addition, GLP-1 effects on GnRH secretion have been explored in ovariectomized ewe models in which GnRH and luteinizing hormone (LH) secretion were suppressed by estrogen and progesterone administration [108]. Collectively, these endocrine and gastrointestinal hormone changes support the concept that gut–brain signaling and adipose tissue remodeling contribute to improved ovulatory function and fertility-related parameters in women with obesity following bariatric surgery [98,99].

Mechanistically, obesity may impair reproductive function through a neuroinflammatory–neuroendocrine pathway: hypothalamic inflammation and microglial activation disrupt central insulin/leptin signaling and may suppress GnRH/LH activity, while pro-inflammatory adipokines and stress-related HPA-axis activation further amplify this adverse central milieu. Together, these changes may promote anovulation, menstrual irregularity, and subfertility, particularly in women with PCOS [27,109].

### 2.5. Pregnancy Recommendations Following Bariatric Surgery

The American College of Obstetricians and Gynecologists (ACOG), the Obesity Society (TOS), and the American Association of Clinical Endocrinology (AACE) advise delaying conception for 12–24 months following BS [110]. This recommended interval allows the attainment of maximal and more stable postoperative weight reduction and is intended to minimize the risk of micronutrient deficiencies and fetal exposure to the rapid catabolic phase, which typically occurs during the first 6–18 months after surgery [40].

In specific clinical circumstances, including advanced maternal age or evidence of reduced ovarian reserve, a shorter surgery-to-conception interval may be considered; however, such decisions require individualized risk–benefit assessment, balancing potential reproductive advantages of earlier conception against the risks of nutritional insufficiency and persistent obesity-related comorbidity [82].

## 3. Discussion

The present review examines the effects of VSG and RYGB on fertility-related outcomes in women with obesity-associated infertility. Obesity is linked to menstrual cycle disturbances, anovulation, and reduced ovarian reserve [24,25]. For women in whom lifestyle intervention and pharmacological therapy do not achieve adequate clinical benefit, bariatric surgery represents an important therapeutic strategy. Beyond metabolic improvement, these procedures are associated with clinically relevant benefits in menstrual regularity, ovulatory function, and overall reproductive outcomes [14].

Our synthesis is in accordance with the prior literature indicating that bariatric surgery is associated with improved menstrual cyclicity, the attenuation of hyperandrogenism, and increased likelihood of conception in women with obesity, particularly in those with PCOS [20]. Clinical reviews have also underscored the importance of postponing conception after surgery and implementing structured nutritional assessment and supplementation during pregnancy [21]. Extending these observations, the present review emphasizes procedure-specific differences between SG and RYGB with respect to reproductive endocrine trajectories (including AMH, SHBG, and androgen measures) and integrates these biochemical changes with clinically relevant fertility endpoints. In particular, the present review addresses the ongoing uncertainty regarding postoperative AMH patterns and discusses biologically plausible explanations and counseling implications, without assuming that AMH changes necessarily correspond to uniform alterations in ovarian reserve or reproductive potential.

VSG is widely performed and is associated with clinically meaningful improvements in weight status, menstrual cycle restoration, and fertility-related outcomes. Available evidence suggests that postoperative weight loss after SG is accompanied by favorable changes in endocrine parameters, including reductions in androgen levels and, in some studies, changes in AMH, including in women with PCOS. These findings are consistent with reports that both SG and RYGB reduce hyperandrogenism and are associated with improved ovulatory function [44,65,73,83]. Notably, improvement in menstrual disturbances may occur within the first months after surgery, supporting a close association between early postoperative weight reduction and reproductive function [44].

RYGB also demonstrates substantial efficacy in improving reproductive and metabolic parameters, particularly among women with severe obesity and menstrual dysfunction; some studies report higher rates of improvement after RYGB compared with VSG, potentially reflecting greater metabolic correction [65,66]. Evidence indicates that laparoscopic RYGB is associated with marked excess weight loss and reductions in testosterone at 6 and 12 months, consistent with improvement in the endocrine–metabolic profile of women with PCOS [56]. Postoperative weight loss after RYGB has been linked to improvement in PCOS-related manifestations, and high conception rates have been reported among infertile women with morbid obesity and PCOS desiring pregnancy [63]. Nevertheless, despite consistent metabolic and clinical improvements, the effects of RYGB on ovarian reserve biomarkers, including AMH, remain incompletely defined [53,55].

Comparison of SG and RYGB indicates both shared and distinct benefits regarding reproductive function. Over follow-up periods of at least 5 years, both approaches can produce substantial and sustained weight reduction and improve obesity-associated comorbidities [50]. Although both procedures are associated with the restoration of menstrual regularity and increased conception rates [63,77,78], SG may be associated with earlier normalization of cycle characteristics and reductions in androgen levels, particularly in women with PCOS [44,83]. In contrast, RYGB has been linked with pronounced postoperative improvements in ovulatory function and fertility outcomes, especially in women with severe obesity [65,66,111]. However, RYGB is more frequently associated with micronutrient deficiencies and increased risk of small-for-gestational-age (SGA) births, whereas VSG generally confers a lower nutritional risk profile while still achieving clinically meaningful weight loss [65,112,113]. Accordingly, procedure selection should be individualized based on patient characteristics, metabolic and endocrine comorbidities, capacity for nutritional follow-up, and reproductive goals.

The interval between bariatric surgery and conception constitutes an additional clinical determinant of outcomes. The first 12–24 months after surgery are characterized by rapid weight loss and potential nutritional fluctuations, which may adversely affect maternal–fetal health and may influence ART outcomes. Consequently, most professional recommendations advise avoiding pregnancy, including ART, for at least 12–18 months after VSG or RYGB to allow stabilization of weight and nutritional status [114]. Conversely, delaying conception in women of advanced reproductive age may reduce fertility potential due to age-related decline in oocyte quality [55,114]. Therefore, counseling should be individualized and coordinated between bariatric and reproductive specialists to balance reproductive timing with postoperative metabolic and nutritional optimization; the optimal surgery-to-conception interval remains insufficiently defined due to limited evidence.

Long-term weight maintenance is also clinically relevant, as postoperative weight regain may adversely affect metabolic status, fertility, and ART outcomes. Available evidence suggests that weight regain may occur within 2–3 years after surgery (approximately 5–10% of lost weight), with larger regain (10–25%) reported by 5–10 years, particularly after VSG [112]. Beyond 7–10 years, substantial regain has been reported in a proportion of patients, reaching 25–30% of the initially lost weight [115,116,117,118,119]. After VSG, approximately 15–25% of patients reportedly regain at least 10% of their postoperative nadir weight within 5 years, whereas after RYGB, although regain may be less frequent, 10–20% may regain >20% of total weight lost within 5–7 years. Across larger cohorts and meta-analyses, clinically significant regain, often defined as regaining >25% of initial weight lost, has been reported in approximately 20–35% of patients within 5–10 years [115,116]. Weight regain may be accompanied by recurrence of anovulation and metabolic dysregulation, potentially attenuating reproductive benefits achieved after surgery. Although RYGB may provide more durable weight loss and metabolic effects than VSG, it carries a higher risk of nutritional deficiencies that may adversely affect fertility and pregnancy outcomes if not rigorously monitored [113].

Maternal mortality after bariatric surgery is infrequently reported. Population-based evidence suggests that bariatric surgery does not increase maternal mortality risk and may reduce it in high-risk women with obesity [65]. Reported absolute maternal mortality risk remains very low (<0.1%, i.e., fewer than 1 per 1000 births) [112,120]. Nonetheless, careful monitoring before and during pregnancy after bariatric procedures is required to mitigate obstetric risk. Interpretation of available data warrants caution, as few studies isolate maternal mortality specifically and much of the literature focuses on composite maternal morbidity, perinatal outcomes, or infant mortality, with potential confounding.

VSG and RYGB are effective interventions for achieving clinically meaningful weight loss and improving fertility-related outcomes in women with obesity, with concomitant improvements in pregnancy-related risks. Optimal reproductive outcomes depend on preconception assessment and continued postoperative nutritional management to support maternal and fetal health. Table 3 summarizes comparative clinical, hormonal, and fertility outcomes for VSG versus RYGB in women with obesity, with and without PCOS, based on the studies reviewed.

This narrative review has several limitations. First, the analysis is restricted to two bariatric procedures (RYGB and SG), which may reduce applicability to other operations with distinct metabolic and nutritional effects (e.g., adjustable gastric banding or biliopancreatic diversion/duodenal switch). Second, despite a structured literature search, the available evidence is predominantly observational and retrospective, with substantial heterogeneity in study populations (PCOS versus non-PCOS cohorts, baseline BMI, and age), follow-up duration, and operational definitions of major outcomes (e.g., “menstrual regularity,” “PCOS remission,” “time to conception,” and fertility endpoints). In addition, several studies rely on self-reported reproductive outcomes and are vulnerable to selection bias, residual confounding (including lifestyle modification and concurrent medical therapies), and limited or absent control groups, collectively limiting causal inference and complicating direct comparison between SG and RYGB.

Another limitation relates to the use of surrogate endocrine biomarkers, particularly AMH, as indicators of ovarian reserve and reproductive potential. Reported AMH patterns after bariatric surgery are inconsistent and may reflect altered follicular dynamics during rapid weight loss or metabolic/endocrine normalization rather than true diminution of ovarian reserve; importantly, few studies longitudinally correlate AMH changes with clinically meaningful endpoints such as ovulation frequency, time to pregnancy, or live birth. Moreover, outcomes relevant to ART (including ovarian response, embryo development, implantation, miscarriage, and live birth) are not consistently reported and are frequently underpowered for procedure-specific comparisons [53,55].

Evidence on long-term reproductive outcomes is also limited. Although some cohorts include follow-up beyond 5 years, high-quality data on fertility, endocrine trajectories, and pregnancy outcomes beyond 5–10 years remain sparse, and the influence of postoperative weight regain on durability of reproductive benefit is insufficiently defined. Weight regain patterns vary by procedure and patient-level factors, and the extent to which recurrent insulin resistance and/or hyperandrogenism contributes to relapse of anovulation or diminished ART success warrants further investigation.

Finally, guidance regarding the optimal surgery-to-conception interval is largely based on expert consensus rather than definitive outcome-driven evidence and requires individualized decision-making to balance nutritional stabilization against age-related decline in fertility. Future large, prospective, multicenter studies using standardized reproductive endpoints (including live birth), consistent outcome definitions, and extended follow-up, while accounting for nutritional status, supplementation adherence, PCOS phenotype, and weight regain trajectories, are needed to strengthen evidence-based counseling and procedure selection for women pursuing fertility after bariatric surgery.

## 4. Methods

This narrative review was prepared in accordance with core quality principles recommended for narrative reviews (SANRA: Scale for the Assessment of Narrative Review Articles) [122], emphasizing a clearly defined scope, transparent literature identification, balanced evidence selection, and structured synthesis.

### 4.1. Review Scope and Outcomes of Interest

The review aimed to summarize and contextualize evidence on the effects of bariatric surgery on female reproductive function and fertility. Outcomes of interest included: (i) menstrual regularity and ovulatory function; (ii) endocrine and metabolic parameters relevant to reproduction (i.e., insulin resistance, androgens, SHBG, gonadotropins); (iii) ovarian reserve markers, particularly AMH; and (iv) fertility-related clinical outcomes such as time to conception, spontaneous conception, assisted reproduction (IVF/ART), pregnancy rates, and live birth where available. The review focused primarily on SG and RYGB, as these represent the most commonly performed procedures worldwide.

### 4.2. Data Sources and Search Strategy

A targeted literature search was conducted in PubMed, Scopus, and Web of Science to identify studies published from database inception to 30 November 2025. Searches were restricted to English-language publications. Search strings combined terms for female fertility and reproductive outcomes with bariatric surgery terms, using Boolean operators. Fertility-related concepts included: fertility, female fertility, reproductive function, ovulation, IVF, assisted reproduction/ART, AMH, polycystic ovary syndrome/PCOS, pregnancy, live birth, time to conception, female obesity, and female infertility. Bariatric surgery concepts included: metabolic surgery, bariatric surgery, weight loss surgery, Roux-en-Y gastric bypass/RYGB, sleeve gastrectomy/SG, and vertical sleeve gastrectomy/VSG.

To improve completeness, the reference lists of included articles and relevant reviews were also screened (“snowballing”) to identify additional eligible studies not captured in the database searches.

### 4.3. Study Selection and Eligibility Criteria

Study selection was performed in two stages. First, titles and abstracts were screened for relevance to bariatric surgery and female reproductive outcomes. Second, potentially relevant articles were reviewed in full text. The following study designs were considered: prospective and retrospective observational studies (cohorts, case–control studies, cross-sectional studies), interventional studies when available, and systematic reviews/meta-analyses used primarily to contextualize findings and identify additional primary studies.

Studies were eligible if they:

- Included women of reproductive age with overweight/obesity;
- Evaluated bariatric surgery—primarily SG and/or RYGB;
- Reported at least one fertility-related outcome (menstrual cyclicity, ovulation, hormonal/metabolic parameters, AMH, conception, ART outcomes, pregnancy and live birth outcomes).

Non-English publications, animal-only studies, case reports with insufficient outcome detail, and studies not reporting reproductive or fertility-relevant endpoints in women after bariatric surgery were excluded.

### 4.4. Data Extraction and Synthesis Approach

Given the heterogeneity of populations (PCOS vs. non-PCOS, baseline BMI, age), surgical techniques, follow-up duration, and outcome definitions across studies, evidence was synthesized narratively rather than through quantitative pooling. For each eligible study, key descriptive and outcome variables relevant to the scope of this review were extracted, including sample size, participant characteristics (age, BMI, PCOS status where reported), bariatric procedure type, follow-up duration, weight loss metrics (%EWL, %TWL), and reproductive outcomes (clinical and biochemical) were extracted. Findings were organized thematically (procedure-specific effects of SG vs. RYGB; AMH and ovarian reserve markers; hormonal/metabolic changes; and fertility/pregnancy outcomes) and summarized in evidence tables to facilitate comparison across studies.

### 4.5. Guidelines and Consensus Documents

In addition to primary studies, clinical guidance and position statements relevant to bariatric surgery and reproductive health were reviewed. Sources included publications from the International Federation for the Surgery of Obesity and Metabolic Disorders (IFSO), the British Obesity and Metabolic Surgery Society (BOMSS), and the American Society for Metabolic and Bariatric Surgery (ASMBS), as these documents inform clinical counseling on timing of pregnancy, contraception, and postoperative nutritional monitoring.

**Figure 3:**
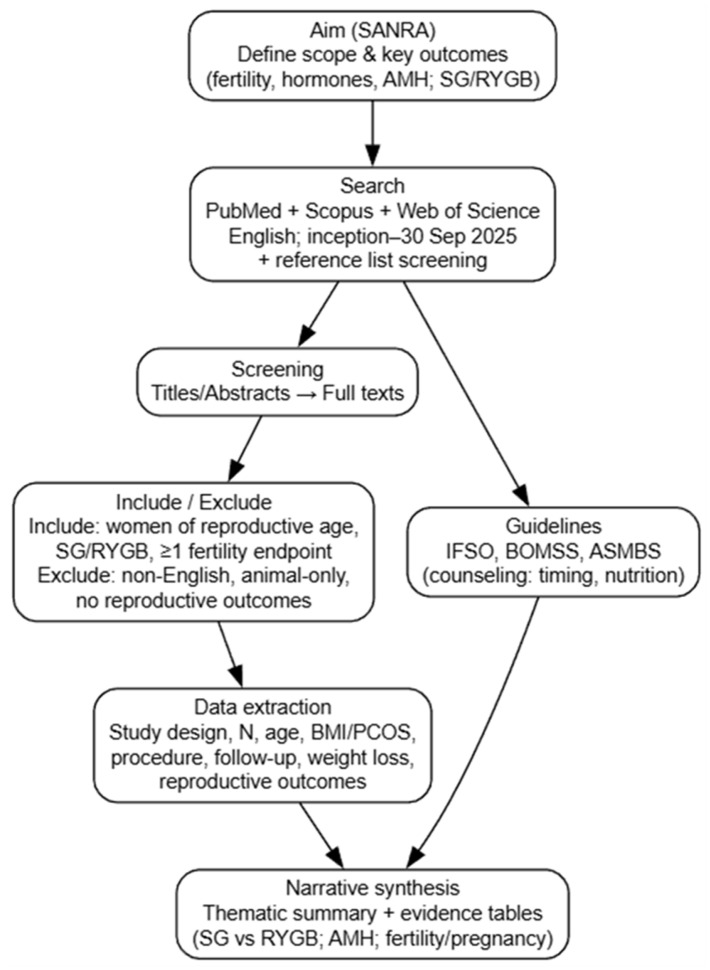
Flow diagram of SANRA-informed narrative review methodology.

We summarized the SANRA-informed narrative review methodology in a flow diagram created with Graphviz (DOT language), illustrating the search, screening, eligibility assessment, data extraction, and thematic narrative synthesis steps (including guideline screening) (Figure 3).

## 5. Conclusions

Available evidence indicates that both VSG and RYGB are associated with improved fertility-related outcomes in women with obesity-associated infertility. These effects extend beyond weight reduction and are likely mediated by interconnected endocrine and molecular adaptations, including the normalization of hypothalamic–pituitary–ovarian axis activity (with improved menstrual cyclicity), enhanced insulin sensitivity, attenuation of chronic low-grade inflammation, and higher pregnancy rates. The selection of the surgical approach should be individualized based on reproductive priorities, cardiometabolic risk profile, and the feasibility of long-term nutritional monitoring.

However, despite increasing clinical experience, direct comparative evidence regarding procedure-specific effects of VSG versus RYGB on ovarian reserve, folliculogenesis, and long-term reproductive outcomes remains limited. Accordingly, large, prospective, multicenter studies comparing VSG and RYGB using standardized reproductive endpoints are required to define the optimal surgical strategy. Such studies should also delineate the most appropriate postoperative interval to conception and characterize longer-term endocrine trajectories to support evidence-based fertility counseling and management in women after bariatric surgery.

## Figures and Tables

**Table 3:** Comparison of VSG vs. RYGB in obese women of reproductive age.

Parameter	SG	RYGB	References
Clinical Guidance for Women Planning Pregnancy	Lower nutritional risk; preferred in women with prioritizing pregnancy.	Requires more intensive nutritional monitoring; preferred in women with severe obesity or metabolic comorbidity (e.g., T2DM).	ASMBS Guidelines; Shehata N et al., 2025 [65]
Weight Loss Metabolic Outcomes	Effective and sustained weight loss; somewhat less than RYGB in long-term studies. Improves insulin sensitivity and metabolic profile.	Greater and more durable weight loss compared with VSG. Stronger metabolic effect, especially in T2DM and severe obesity.	Johansson et al., 2015 [113] Akhter et al., 2019 [112]
Fertility Outcomes (Ovulation, Menstrual Cycles, PCOS)	Improved menstrual regularity, restoration of ovulation, reduced hyperandrogenism. Improving fertility in PCOS obese women.	Similar or stronger effect on ovulation and fertility improvement, particularly in women with severe obesity.	Goldman 2016 [111] Shehata et al., 2025 [65]
Spontaneous Conception Rates	Increased spontaneous conception rates after weight loss. Limited long-term comparative data.	Increased conception rates, sometimes higher than SG in observational studies.	Shehata et al., 2025 [65]
Assisted Reproduction Outcomes (IVF)	Higher ovarian response rates, improved oocyte quality after weight loss, enhanced endometrial receptivity, ART positive results.	Similar benefits; however, micronutrient status may affect ART outcomes.	Goldman et al., 2016 [111] Rittenberg et al., 2011 [121]

## Data Availability

No new data were created or analyzed in this study. Data sharing is not applicable to this article.

## Abbreviations

The following abbreviations are used in this manuscript:

AACE	The American Association of Clinical Endocrinology
ACOG	The American Congress of Obstetricians and Gynecologists
AMH	Anti-Müllerian Hormone
ART	Assisted Reproductive Technology
ASMBS	The American Society for Metabolic and Bariatric Surgery
ASRM	The American Society of Reproductive Medicine Committee
BL	Baseline
BR	Birth Rate
BS	Bariatric Surgery, often called MBS: Metabolic and Bariatric Surgery
BMI	Body Mass Index
BSCI	Bariatric Surgery to Conceptional Interval
CHD	Coronary Heart Disease
CVD	Cardiovascular Disease
DHEA	Dehydroepiandrosterone sulfate
ΔBMI	“Change” in Body Mass Index
EWL	Excess Weight Loss
FAI	Free Androgen Index
FSH	Follicle-Stimulating Hormone
GDM	Gestational Diabetes Mellitus
GNRH	Gonadotrophin-Releasing Hormone
HFD	High-Fat Diet
HPO axis	Hypothalamic–Pituitary–Ovarian axis
IFSO	The International Federation for the Surgery of Obesity and Metabolic Disorders
IR	Insulin Resistance
IUD	Intrauterine Device
IVF	In Vitro Fertilization
LARC	Long-Acting Reversible Contraception
LBW	Low Birth Weight
LGA	Large-for-gestational-age
LH	Luteinizing Hormone
LMT	Lifestyle Modification Therapy
MCI	Menstrual Cycle Irregularities
NC	Natural Conception
NICE	National Institute for Health and Clinical Excellence
OR	Odds Ratio
OCPs	Oral Contraceptive Pills
PCOS	Polycystic Ovarian Syndrome
RYGB	Roux-en-Y Gastric Bypass, often called LYRGB (Laparoscopic RYGB)
SG	Sleeve Gastrectomy, often called LSG (Laparoscopic SG) or VSG (Vertical Sleeve Gastrectomy)
SGA	Small for Gestational Age
SHBG	Sex Hormone-Binding Globulin
T2DM	Type 2 Diabetes Mellitus
TOS	The Obesity Society
TWL	Total Weight Loss
VAT	Visceral Adipose Tissue
VLCD	Very Low-Calory Diet
WHO	World Health Organization
